# Assessment of Pregnant Women’s Satisfaction With the Model of Care Initiative: Antenatal Care Services at Primary Health Care Centers in the Qassim Health Cluster, Saudi Arabia

**DOI:** 10.7759/cureus.76383

**Published:** 2024-12-25

**Authors:** Ghaday Almutairi, Layan S Alshmrani, Rahaf Mishal Alomairi, Mohammed S Alotaibi, Norah H Alhumaidi, Rayan Muslih Alotaibi, Shahad Mubarak Aljebeli, Suha Ali Alarfaj, Shaden Ali Alhenaki, Bashaer Abdulaziz Albedah, Tameem A Alhomaid

**Affiliations:** 1 Medicine, Unaizah College of Medicine and Medical Sciences, Qassim University, Qassim, SAU; 2 Medicine, King Khalid University, Abha, SAU; 3 Family Medicine, Collage of Medicine, Batterjee Medical College, Jeddah, SAU; 4 College of Medicine, Alexandria University, Alexandria, EGY; 5 Obstetrics and Gynecology, College of Medicine, Alexandria University, Alexandria, EGY; 6 College of Medicine, King Saud University for Health and Sciences, Riyadh, SAU; 7 Family Medicine, Qassim University, Qassim, SAU; 8 Family Medicine, ⁠King Fahad Specialist Hospital, Buraydah, SAU; 9 Family Medicine, Qassim Health Cluster, Buraydah, SAU

**Keywords:** antenatal care, healthcare quality, maternal health, patient satisfaction, qassim health cluster, saudi arabia

## Abstract

Background: Patient satisfaction is a critical indicator of healthcare quality, including high-quality antenatal care (ANC), and it directly impacts care continuity and health outcomes. This study assessed the satisfaction levels of pregnant women with ANC services provided at primary healthcare centers (PHCs) within the Qassim Health Cluster, Saudi Arabia.

Methods: A cross-sectional survey was conducted among 646 pregnant women attending ANC services in the Qassim region. Participants completed a validated questionnaire assessing demographic characteristics, healthcare interactions, and satisfaction levels across various ANC service aspects, including waiting times, healthcare provider communication, and healthcare service quality. Statistical analyses, including Pearson’s chi-squared tests, Fisher’s exact tests, and regression analyses, were performed to identify predictors of satisfaction.

Results: The majority of participants (N=366, 90.6%) reported successfully booking their appointments, and 368 (91.1%) faced no challenges during the process. While 393 (97.3%) and 398 (98.5%) reported receiving weight and blood pressure measurements, respectively, only 312 (77.2%) were advised on diet, and 298 (73.8%) received explanations about alarming symptoms. Communication gaps were noted, with 71% (N=287) of participants indicating that physicians did not introduce themselves, and 307 (76%) reported that physicians did not disclose their specialties. Satisfaction levels were highest for maintaining privacy (273 (67.6%) highly satisfied) and healthcare staff attitudes (247 (61.1%) highly satisfied), with an overall satisfaction mean score of 4.31±0.869. Regression analyses identified healthcare service levels and years of marriage as significant predictors of satisfaction (p<0.05).

Conclusion: While overall satisfaction with ANC services was high, areas such as physician communication and health education require improvement. Interventions focusing on enhancing patient-provider interactions, providing comprehensive health education, and optimizing service delivery could further improve satisfaction.

## Introduction

Appropriate prenatal care plays a critical role in promoting maternal and fetal health [1]. It encompasses a range of interventions, including health education on parenthood and family life, therapy, screening, and counseling, all of which contribute to mitigating pregnancy-related complications [2,3]. High-quality antenatal care (ANC) is essential for the early identification and management of risk factors that may jeopardize pregnancy outcomes. Globally recognized as a fundamental preventive health service [4], ANC is vital for ensuring the well-being of pregnant women and their unborn children, whether provided in hospitals or through primary healthcare centers (PHCs) [2,4]. To minimize maternal and neonatal morbidity and mortality, ANC practitioners are required to identify and manage risks proactively while delivering comprehensive health education. The World Health Organization (WHO) and other international standards have historically recommended a minimum of four ANC visits during pregnancy [3]. However, in 2016, the WHO revised its guidelines, advocating for eight antenatal contacts to improve maternal and neonatal outcomes [3,5].

Patient satisfaction is a pivotal measure in assessing and enhancing the quality of healthcare services. It has increasingly been recognized as a critical component of ANC improvement initiatives [6]. Understanding and integrating pregnant women’s feedback into healthcare planning ensures continuous patient monitoring and fosters a positive relationship between patients and healthcare providers [7]. Satisfied patients are more likely to develop stronger, lasting bonds with their providers, enhancing continuity of care, compliance, and health outcomes [8]. Studies have shown that inadequate ANC coverage correlates with higher maternal mortality rates [9,10]. Poor quality ANC negatively affects maternal perceptions and satisfaction, with issues like unclean or uncomfortable waiting areas contributing to dissatisfaction [11,12].

In Saudi Arabia, a study evaluating pregnant women’s satisfaction with ANC services at PHCs in Riyadh revealed varying levels of satisfaction: 93.7% with initial triage assessment, 87.8% with provided services, 71.8% with consultations, and 53.9% with examinations [13]. These findings highlight the need for improvements in consultation and examination processes to boost overall satisfaction. Given the importance of patient satisfaction in driving healthcare improvements, this study aimed to assess the level of satisfaction among pregnant women receiving ANC services at PHCs within the Qassim Health Cluster, Saudi Arabia. By identifying areas of strength and opportunities for enhancement, the study seeks to inform strategies for optimizing maternal healthcare delivery.

## Materials and methods

Study site and design

This study was conducted at PHCs within Buraidah, Al-Qassim region. PHCs with established ANC services were selected to ensure accessibility for pregnant women. Additionally, advanced healthcare facilities and secondary health facilities in the selected region were identified to provide comprehensive coverage of ANC services. The study employed a cross-sectional survey design, using a self-administered online and on-site questionnaire given to all pregnant women attending health care facilities for ANC in Buraidah, Al-Qassim region.

Population

The inclusion criteria for eligible participants were as follows: pregnant females receiving follow-up care at the aforementioned facilities, having attended at least one ANC visit during the current pregnancy, and capable of completing the questionnaire in either Arabic or English. We excluded pregnant women who receive follow-up care in private hospitals or PHCs without ANC clinics, as well as non-pregnant female patients.

Sample size

We used OpenEpi to calculate the necessary sample size to be 384 participants, at minimum, aiming to achieve a confidence level of 95% with a precision of ±0.05 in addition to the proportion or prevalence (p) set at 0.5 due to the unknown proportion. However, considering the utilization of cluster and stratified sampling techniques, a design effect of 1.5 was applied [14], leading to 576 as a sample (384×1.5=576 participants). Additionally, accounting for an anticipated non-respondent rate of 8%, the total enrollment became 646 participants (384×1.5+70).

Sampling technique 

Proportionate stratified sampling was employed based on the rate of ANC attendance at selected PHCs during the specified timeframe. This approach ensured representation from various demographic groups among pregnant women seeking ANC services.

Data collection methods, instructions used, and measurements

A validated English questionnaire was adopted for data collection, with necessary translations into Arabic. Prior to the main data collection, a pilot study was conducted with a small sample of pregnant women to assess the reliability and internal consistency of the questionnaire. Amendments were made based on the pilot study results. The questionnaire captured sociodemographic variables, including age, education level, monthly income, number of previous pregnancies, and ANC visit history. Clinical data such as weight, height, blood pressure, blood test results, ultrasound findings, urine test results, and medication history were also recorded. Additionally, feedback on participants' preferences and satisfaction with ANC services, including waiting times, comfort, privacy, and perceived qualifications of the healthcare team, were collected. 

During data collection, trained personnel assisted participants in completing online questionnaires at the healthcare facilities and on social media. 

Data management and analysis plan

Data completeness and accuracy were ensured through thorough review and double-checking during data entry. Statistical analysis was conducted using R statistical software. Descriptive statistics were used to summarize the demographic characteristics of the study population and the distribution of key variables. Continuous variables were presented as mean ± standard deviation or median with interquartile range, while categorical variables were expressed as frequencies and percentages. Inferential statistical analyses were conducted to explore associations and identify predictors of satisfaction with ANC services. Specifically, Fisher’s exact tests and Pearson’s chi-squared analyses were performed for categorical variables, while the Shapiro-Wilk test was used to assess the normality assumptions of continuous variables. To determine satisfaction levels, mean values were used as cutoff points, with satisfaction dichotomized into adequate (coded as 1) and inadequate (coded as 0). ANOVA analysis was performed to compare satisfaction levels across different biographical variables. Simple linear regression analyses were employed to examine the influence of various factors, such as sociodemographic characteristics and clinical variables, on satisfaction with ANC services. Furthermore, a more comprehensive analysis was conducted using linear regression models to identify independent predictors of satisfaction while adjusting for potential confounders. Variables found to be statistically significant in the univariate analyses were included in the multivariate models. P-values were set at <0.05 for statistical significance, and confidence intervals were calculated.

Ethical considerations

Ethical approval was obtained from the National Committee of Bio-Ethics (H-04-Q-001). Participation in the study was voluntary, and informed consent was obtained from each participant. Participant anonymity and confidentiality were maintained throughout the study. Participants were also fully informed about the purpose, methods, potential benefits, and risks of the study. Participants had the right to withdraw from the study at any time without consequences. We securely stored the data and kept them accessible only to authorized personnel. Moreover, no physical, psychological, or social harm to participants was done.

## Results

Participant characteristics

Table 1 shows the demographic and biographical characteristics of the study participants. Half of respondents were aged between 31 and 40 years (N=203, 50.2%), and the second largest group was aged 20-30 years (N=141, 34.9%). Most participants had attained at least a bachelor's degree (N=277, 68.6%). 19.8% had been married for seven to 10 years, and the same percentage had been married for more than 15 years. In terms of pregnancy history, a quarter of participants (N=102, 25.2%) had experienced three to five pregnancies, and a significant proportion (N=172, 42.6%) were in their second trimester (14-27 weeks). The frequency of antenatal visits was also assessed, with 42.6% (N=172) of participants attending two to four visits during their current pregnancy.

**Table 1:** Demographic and biographical characteristics of participants

Variables	N	%
Age		
Younger than 20 years	18	4.5%
20-30years	141	34.9%
31-40 years	203	50.2%
Older than 40 years	42	10.4%
Educational level		
Did not receive an education	5	1.2%
Primary school	4	1.0%
Intermediate school	8	2.0%
High school	54	13.4%
Diploma degree	56	13.9%
Bachelor's degree or higher	277	68.6%
How long have you been married?		
1 year	37	9.2%
2-4 years	60	14.9%
5-7 years	73	18.1%
7-10 years	80	19.8%
11-15 years	74	18.3%
More than 15 years	80	19.8%
The number of times you have been pregnant, including this pregnancy		
The first pregnancy	70	17.3%
2 times	68	16.8%
3-5 times	102	25.2%
4 times	88	21.8%
More than 5 times	76	18.8%
How long have you been pregnant?		
1-13 weeks (first-third month)	129	31.9%
14-27 weeks (fourth sixth month)	172	42.6%
28-42 weeks (seventh-ninth month)	103	25.5%
During this pregnancy, how many antenatal visits have you had?		
One visit	82	20.3%
2-4 visits	172	42.6%
5-8 visits	90	22.3%
More than 8 visits	60	14.9%

Patient appointment and interaction experience with healthcare providers

Table 2 shows the distribution of responses regarding healthcare providers and appointment-related details for the participants. The majority of respondents reported seeing an obstetrician/gynecologist (N=155, 38.4%) or a family physician (N=103, 25.5%). Notably, 90.6% (N=366) of participants confirmed that their appointment was booked in advance, while a small proportion (N=38, 9.4%) did not have a booked appointment. Additionally, most participants (N=368, 91.1%) did not face difficulty with appointment booking, although a few reported issues such as congestion or problems with the Sehaty app. Regarding the physician's introduction, a significant percentage (N=10, 71%) of participants indicated that the physician did not introduce themselves, and 307 (76%) reported that the physician did not introduce their specialty. The waiting time after vital signs were taken varied; the higher time was a five- to ten-minute wait, reported by 142 (35.1%) participants.

**Table 2:** Patient appointment and interaction experience with healthcare providers

Items	N	%
Health care provider		
Nurse	1	0.2%
Family physician	103	25.5%
General practitioner	81	20.0%
Obstetrics and gynecology	155	38.4%
I don't know	64	15.8%
Is this appointment booked?		
No	38	9.4%
Yes	366	90.6%
Have you had difficulty booking your appointment?		
No	368	91.1%
I didn't book an appointment	22	5.4%
Yes	14	3.5%
The reason for the difficulty		
Congestion and difficulty getting an appointment	10	71.4%
Difficulty booking through the Sehaty app	4	28.6%
Time of waiting after taking the vital signs, by physician		
Less than 5 minutes	58	14.4%
5-10 minutes	142	35.1%
11-20 minutes	102	25.2%
21-30 minutes	65	16.1%
More than 30 minutes	37	9.2%
Did the physician introduce his/her name to you?		
No	287	71.0%
Yes	117	29.0%
Did the physician introduce his/her specialty to you?		
No	307	76.0%
Yes	97	24.0%

Healthcare services provided for follow-up

Table 3 shows data on the healthcare services provided to participants during their pregnancy. The majority of respondents reported that essential health measurements such as weight (N=393, 97.3%), height (92.3%), and blood pressure (N=398, 98.5%) were measured during their pregnancy. Furthermore, 95% (N=384) of participants received supplements such as folic acid, iron, or calcium. However, fewer participants were advised on diet (N=312, 77.2%) or instructed on medication use (N=360, 89.1%). Over a quarter (N=106, 26.2%) of respondents reported that alarming symptoms were not explained to them during the pregnancy. Regarding services in the last three months of pregnancy, 168 (57.9%) participants discussed contraception with their healthcare provider, and 183 (63.1%) discussed breastfeeding. The majority (N=285, 70.5%) had the opportunity to choose where to deliver their baby. Additionally, the result revealed that most consultations lasted less than 15 minutes (N=250, 61.9%), and the overall healthcare service score, based on participant feedback, had a mean score of 7.80±2.18 out of 10.

**Table 3:** Healthcare services provided for follow-up during pregnancy

Items	No		Yes	
	N	%	N	%
During this pregnancy, were you weighed?	11	2.7%	393	97.3%
During this pregnancy, was your height measured?	31	7.7%	373	92.3%
During this pregnancy, was your blood pressure measured?	6	1.5%	398	98.5%
During this pregnancy, were the supplements like "folic acid, iron, or calcium" prescribed to you?	20	5.0%	384	95.0%
During this pregnancy, were you advised about your diet?	92	22.8%	312	77.2%
During this pregnancy, were you instructed on how to use your medications?	44	10.9%	360	89.1%
During this pregnancy, were alarming symptoms explained to you?	106	26.2%	298	73.8%
During the last 3 months of pregnancy, did the health practitioner discuss contraception with you? (N=290)	122	42.1%	168	57.9%
During the last 3 months of pregnancy, did the health practitioner discuss breastfeeding with you? (N=290)	107	36.9%	183	63.1%
During this pregnancy, did you have the chance to choose where to deliver your baby?	119	29.5%	285	70.5%
How long does the consultation last?	No		Yes	
Less than 15 minutes	250		61.9%	
More than 15 minutes	154		38.1%	
Healthcare service score (out of 10)	Mean ± SD		7.80±2.18	

**Figure 1:**
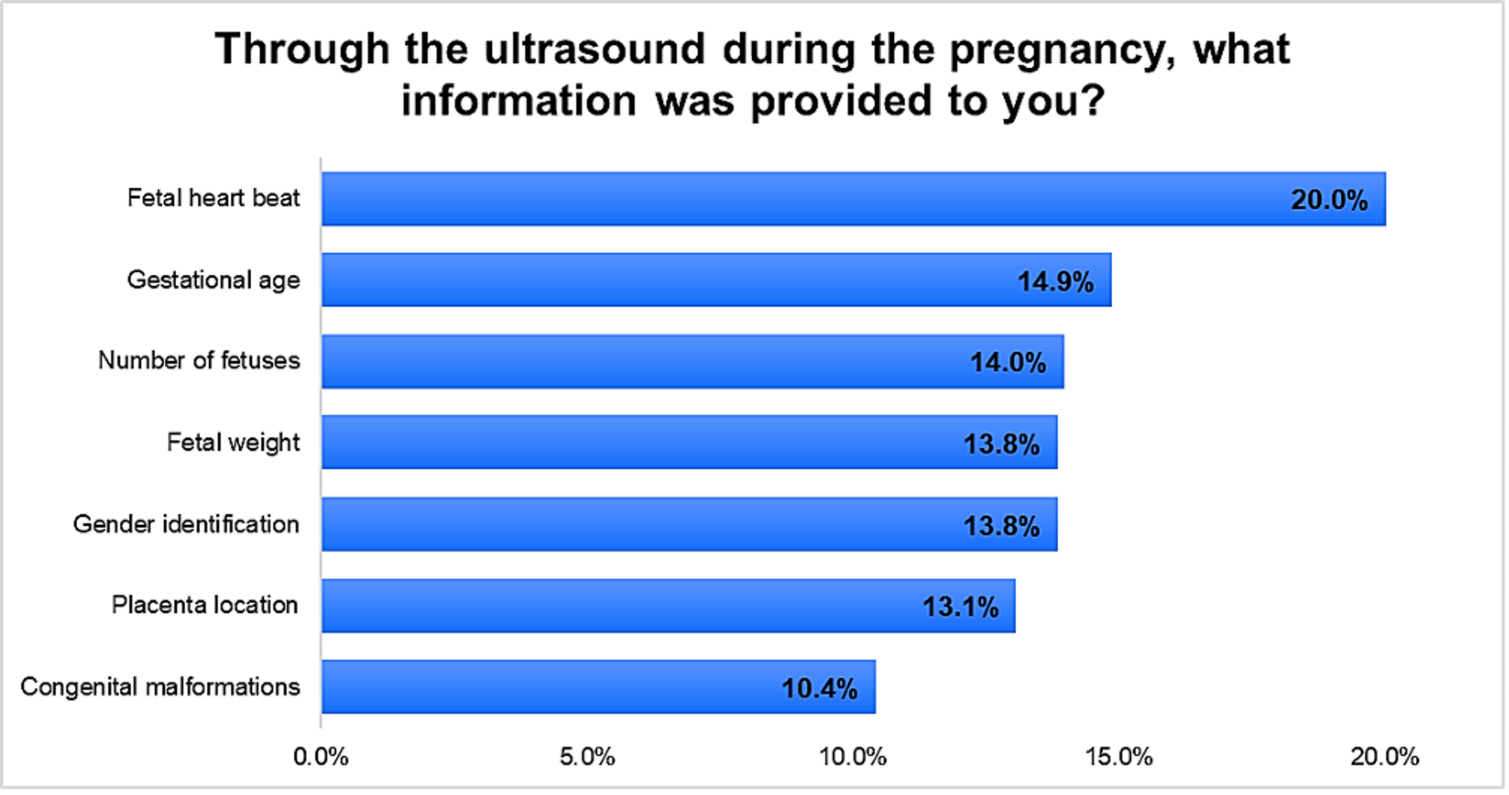
Information received by participants from ultrasound

**Figure 2:**
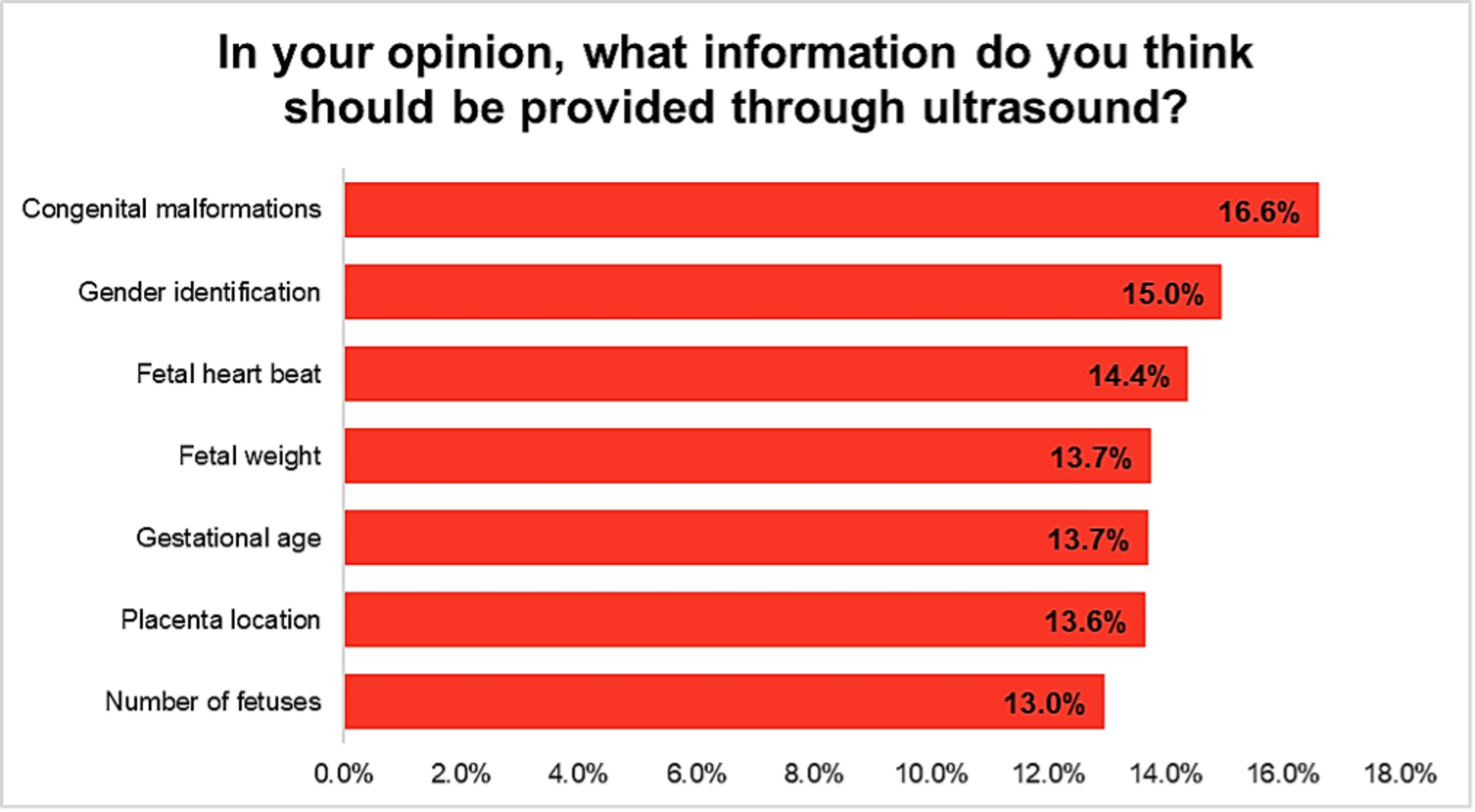
Participants’ expectations of information provided through ultrasound

Figure 1 and Figure 2 illustrate the distribution of responses regarding the information participants expect to be provided through ultrasound during pregnancy as well as the information that participants received from ultrasound during pregnancy. The highest percentage of respondents (16.6%) emphasized the importance of detecting congenital malformations. Gender identification followed closely at 15.0%, while fetal heart rate monitoring was considered important by 14.4% of participants. Other crucial data included fetal weight (13.7%), gestational age (13.7%), placenta location (13.6%), and the number of fetuses (13.0%). Regarding the received information from ultrasound during pregnancy, the most commonly received information was the detection of congenital malformations (16.6%), followed by gender identification (15.0%) and fetal heart rate (14.4%). The remaining information, including fetal weight (13.7%), gestational age (13.7%), placenta location (13.6%), and the number of fetuses (13.0%), were also considered important by the participants. 

Satisfaction level assessment

Table 4 presents the satisfaction levels of participants across various aspects of ANC services. The highest levels of satisfaction were observed in the areas of healthcare staff attitude (N=247, 61.1% highly satisfied), maintaining privacy during the consultation (N=273, 67.6% highly satisfied), and discussing health status with the physician (N=255, 63.1% highly satisfied). Satisfaction with waiting time was moderately distributed, with 200 (49.5%) participants being highly satisfied, while a small proportion, 21 (5.2%), were very unsatisfied with the waiting time. Other aspects, such as the physician’s decision-making (N=258, 63.9% highly satisfied) and the treatment dispensing process (N=266, 65.8% highly satisfied), also received high satisfaction scores. The overall satisfaction mean score was 4.31±0.869, indicating generally positive feedback from the participants on the ANC services provided.

**Table 4:** Satisfaction levels of participants with ANC services SD, standard deviation; ANC, antenatal care

Items		Very Unsatisfied	Unsatisfied	Neutral	Satisfied	Highly satisfied
How satisfied are you with the waiting time?	N	21	49	61	73	200
	%	5.2%	12.1%	15.1%	18.1%	49.5%
How satisfied are you with the attitude of healthcare staff?	N	12	14	41	90	247
	%	3.0%	3.5%	10.1%	22.3%	61.1%
How satisfied are you with maintaining privacy during the consultation?	N	12	16	32	71	273
	%	3.0%	4.0%	7.9%	17.6%	67.6%
How satisfied are you with discussing your health status with the physician?	N	11	12	43	83	255
	%	2.7%	3.0%	10.6%	20.5%	63.1%
How satisfied are you with the physician’s decision-making on your condition?	N	6	11	42	87	258
	%	1.5%	2.7%	10.4%	21.5%	63.9%
How satisfied are you with laboratory working hours to perform the required tests?	N	30	27	52	74	221
	%	7.4%	6.7%	12.9%	18.3%	54.7%
How satisfied are you with healthcare facilities?	N	7	17	51	88	241
	%	1.7%	4.2%	12.6%	21.8%	59.7%
How satisfied are you with the treatment dispensing process?	N	12	11	28	87	266
	%	3.0%	2.7%	6.9%	21.5%	65.8%
How satisfied are you with this visit?	N	10	9	43	86	256
	%	2.5%	2.2%	10.6%	21.3%	63.4%
Overall, how satisfied are you with the antenatal care services provided?	N	13	16	35	84	256
	%	3.2%	4.0%	8.7%	20.8%	63.4%
Satisfaction mean score (out of 5)		Mean ± SD			4.31±0.869	

**Figure 3:**
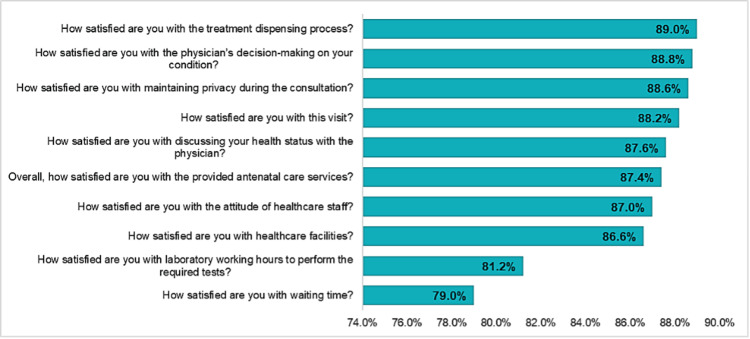
Relative weights of the satisfaction items

Figure 3 shows the relative weights of the satisfaction items ranked in ascending order, ranging from 76.0% to 89.0%. Satisfaction with the treatment dispensing process had the highest score at 89.0%, followed closely by satisfaction with physician decision-making at 88.8% and maintaining privacy during the consultation at 88.6%. Other notable scores included satisfaction with this visit at 88.2% and discussing health status with the physician at 87.6%. The lowest scores were for satisfaction with laboratory working hours for required tests at 81.2% and waiting time at 79.0%.

Comparison of satisfaction levels regarding biographical data of the participants

Table 5 shows the results of an ANOVA analysis comparing satisfaction levels across different biographical variables. Significant differences were found in satisfaction levels based on age (F=2.914, p=0.034), educational level (F=3.174, p=0.008), years of marriage (F=4.427, p=0.001), and number of ANC visits (F=2.901, p=0.035), indicating that these factors influenced participants’ satisfaction with ANC services. In contrast, no significant differences were found based on the total number of pregnancies (F=0.453, p=0.770), or duration of pregnancy (F=0.079, p=0.924), suggesting that these factors did not have a significant effect on satisfaction levels.

**Table 5:** Comparison of satisfaction levels by biographical data of participants *Significant at <0.05 **Significant at <0.01 ANC, antenatal care

Variables		Sum of squares	df	Mean square	F	P-value
Age	Between groups	6.51	3	2.171	2.914	0.034*
	Within groups	297.9	400	0.745		
Educational level	Between groups	11.67	5	2.335	3.174	0.008**
	Within groups	292.8	398	0.736		
Years of marriage	Between groups	16.0	5	3.208	4.427	0.001**
	Within groups	288.4	398	0.725		
Total number of pregnancies	Between groups	1.38	4	0.344	0.453	0.770
	Within groups	303.1	399	0.760		
Long time of pregnant	Between groups	0.120	2	0.060	0.079	0.924
	Within groups	304.3	401	0.759		
Number of ANC visits during current pregnancy	Between groups	6.48	3	2.161	2.901	0.035*
	Within groups	297.9	400	0.745		

Correlation between satisfaction levels and healthcare service levels

**Figure 4:**
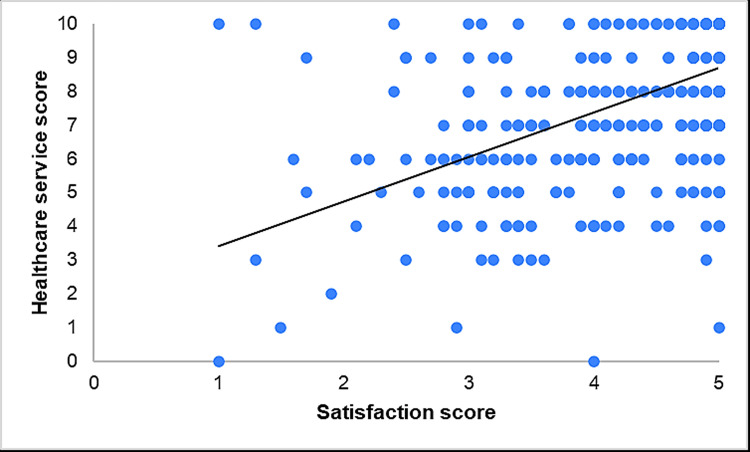
Scatter plot for the relation between satisfaction and healthcare service levels

Table 6 and Figure 4 show the results of the Pearson correlation analysis between participants' satisfaction levels and the levels of healthcare services. A significant positive correlation was found (r=0.527, p<0.001), suggesting that higher satisfaction levels are significantly associated with improved perceptions of healthcare service quality. 

**Table 6:** Correlation between satisfaction levels and healthcare service levels **Significant at <0.01

Items	Pearson correlation	P-value
Satisfaction level	0.527	<0.001**
Healthcare service levels		

Regression analysis model 

Table 7 presents the results of a simple linear regression analysis examining predictors of satisfaction levels. Significant predictors included age (B=-0.158, 95% CI=-0.276 to -0.041, p=0.008), years of marriage (B=-0.110, 95% CI=-0.162 to -0.057, p<0.001), number of ANC visits (B=0.104, 95% CI=0.015 to 0.192, p=0.021), and healthcare service levels (B=0.210, 95% CI=0.176 to 0.243, p<0.001). These results mean that increasing in age and more years of marriage negatively impact pregnant women’s satisfaction with the model of care initiative. Conversely, the increasing number of ANC visits and the higher healthcare service positively impacted the satisfaction of pregnant women. On the contrary, the educational level did not emerge as a significant predictor (B=-0.066, p=0.126). The overall model indicates that healthcare service levels are the most influential factor in predicting satisfaction levels among the participants.

**Table 7:** Simple linear regression model predicting satisfaction level The dependent variable is the satisfaction level. *Significant at <0.05 ** Significant at <0.01 ANC, antenatal care

Predictors	B (95% CI)	Std. Error	t	P-value
Age	-0.158 (-0.276 - -0.041)	0.060	-2.661	0.008**
Educational level	-0.066 (-0.151 - 0.019)	0.043	-1.533	0.126
Years of marriage	-0.110 (-0.162 - -0.057)	0.027	-4.125	<0.001**
Number of ANC visits during current pregnancy	0.104 (0.015 - 0.192)	0.045	2.309	0.021*
Healthcare service levels	0.210 (0.176 - 0.243)	0.017	12.423	<0.001**

Finally, the results of a multiple linear regression analysis to identify predictors of satisfaction levels are shown in Table 8. The predictor variables chosen were the significant variables in the simple regression model, including age, years of marriage, number of ANC visits during current pregnancy, and healthcare service levels. The results revealed that years of marriage (B=-0.078, 95% CI=-0.139 to -0.018, p=0.011) and healthcare service levels (B=0.200, 95% CI=0.167 to 0.234, p<0.001) were significant predictors of satisfaction when keeping other variables constant. Specifically, fewer years of marriage and higher healthcare service levels were associated with higher satisfaction levels. Neither age (B=-0.004, p=0.949) nor the number of ANC visits (B=0.029, p=0.462) significantly predicted satisfaction levels. The overall model was statistically significant (F(4, 399)=42.4, p<0.001) and explained approximately 29.8% of the variance in satisfaction levels (R²=0.298).

**Table 8:** Multiple linear regression model predicting satisfaction level The dependent variable is the satisfaction level. *Significant at <0.05 **Significant at <0.01 ANC, antenatal care

Predictors	B (95% CI)	Std. Error	t	P-value
Age	-0.004 (-0.138 - 0.130)	0.068	-0.064	0.949
Years of marriage	-0.078 (-0.139 - -0.018)	0.031	-2.544	0.011*
Number of ANC visits during current pregnancy	0.029 (-0.048 - 0.106)	0.039	0.737	0.462
Healthcare service levels	0.200 (0.167 - 0.234)	0.017	11.658	<0.001**
F (4,399)=42.4, P-value<0.001. R^2^=0.298				

## Discussion

This study assessed pregnant women’s satisfaction with ANC services in PHCs within the Qassim Health Cluster, Saudi Arabia. The results provide valuable insights into the factors influencing satisfaction and highlight areas for potential improvement in ANC services. In this study, 71% of participants reported that the physician did not introduce themselves, and 76% indicated that the physician did not share their specialty. These figures highlight a gap in patient-physician communication, which is a critical component of patient satisfaction. A study conducted in Riyadh, Saudi Arabia, reported similar communication gaps during consultations, with overall satisfaction with consultation and examination being 71.8% and 53.9%, respectively [13]. Similarly, another study emphasized the importance of effective communication, which significantly impacts patient satisfaction with primary healthcare services [15]. Studies showed that enhancing physician communication skills improved healthcare quality and patient satisfaction [15,16]. The lack of introductions by healthcare providers in this study might indicate an area where cultural or systemic factors are affecting communication. Incorporating mandatory training in communication skills for physicians could address this gap. It was established that training in communication skills positively changes physician's behavior, leading to high patient satisfaction [17]. Training programs focusing on interpersonal skills, cultural competence, and empathy could improve satisfaction levels. Studies have demonstrated that patient satisfaction improves significantly when healthcare providers use communication strategies that build trust and rapport [15,18]. Given the importance of cultural nuances in patient satisfaction, incorporating culturally sensitive practices could enhance trust and communication, and studies have shown that culturally tailored interventions improve disease knowledge, healthcare access, and care coordination and are cost-effective in improving satisfaction among diverse populations [19,20].

In our study, most participants (91.1%) did not face difficulties booking appointments, and 35.1% reported waiting times between five and 10 minutes after their vital signs were taken. Comparatively, the Riyadh study found that waiting time satisfaction was lower, suggesting improvements in the Qassim region's system [13]. In Ethiopia, where longer waiting times were reported, it was found that long waiting times negatively impact satisfaction with ANC services [11,12]​, aligning with studies conducted in China and South Africa [21,22]. This highlights the importance of optimizing clinic workflows to reduce patient wait times further.

The majority of participants in this study reported that essential healthcare services, such as weight, height, and blood pressure measurements, were consistently provided (over 90%). This is consistent with WHO recommendations for quality ANC care [3,23]. However, areas such as dietary advice (77.2%) and explanations of alarming symptoms (73.8%) had lower rates compared to the aforementioned services, indicating room for improvement to optimize pregnancy outcomes. A healthy, balanced diet is vital for positive pregnancy outcomes, and the consumption of such a diet before and during pregnancy is associated with a reduced risk of pregnancy disorders [24,25]. Physicians' education and counseling during pregnancy improve women's nutritional status during pregnancy and early detection of complications, leading to improved outcomes. Interactive methods such as digital platforms and group education sessions have been effective in educating pregnant women. Research showed a significant difference in the total scores of satisfaction with childbirth preparation courses between the virtual group and the in-person training group (P=0.028). All virtual group members (100%) and 46.7% of the in-person group members were satisfied with the educational content [26].

Our findings highlighted healthcare service levels and years of marriage as significant predictors of satisfaction. Higher service levels positively influenced satisfaction, while more years of marriage were associated with lower ANC satisfaction, which might be attributed to familiarity over the years and increasingly high expectations. These findings align with a study conducted in Nigeria, which found that sociodemographic factors, such as marital status, influenced ANC satisfaction​ [27]. Higher satisfaction with high healthcare service levels might be attributed to the presence of more skilled healthcare providers at the higher levels. Evidence shows that patients attending the primary care facilities were less satisfied than patients at higher hospital levels and the private facilities [28,29]. Investigating satisfaction levels between urban and rural populations could highlight specific barriers faced by different groups and inform targeted interventions, and exploring the role of partner involvement in ANC could provide a holistic understanding of satisfaction determinants. Studies have shown that partner support positively impacts maternal health outcomes and satisfaction [30-32].

This study has limitations that need to be acknowledged. These include the cross-sectional nature of the study, which limits its ability to establish causality between predictors and satisfaction levels. The self-reported data from participants introduces the possibility of recall bias and social desirability bias, which could have led to overestimating or underestimating satisfaction levels. The study was conducted exclusively in PHCs within the Qassim Health Cluster, limiting its generalizability to other regions in Saudi Arabia. Potential sampling bias exists, as participants who agreed to complete the survey might have been more likely to have positive or negative experiences, skewing the overall satisfaction levels. Unmeasured variables such as mental health status, cultural beliefs, or previous experiences with healthcare systems could play a significant role in shaping perceptions of ANC services. Therefore, future research should consider conducting longitudinal studies to explore causal relationships, incorporating qualitative methods, expanding the study to include diverse geographic regions and healthcare settings, ensuring a representative sample, investigating additional variables, and including the perspectives of partners and families.

## Conclusions

The study indicates that, while overall satisfaction levels are generally high, with notable strengths in staff attitudes, privacy during consultations, and physician decision-making, several areas warrant improvement, including communication lapses. Factors influencing satisfaction were also identified, with healthcare service levels emerging as a significant positive predictor. Conversely, longer durations of marriage were associated with lower satisfaction, underscoring the need for personalized care that adapts to the varying expectations and experiences of patients. These results underscore the importance of addressing these gaps through targeted interventions, including enhanced communication training for healthcare providers, comprehensive health education programs, and further optimization of appointment systems and service delivery. Future research should explore longitudinal trends, rural-urban disparities, and the integration of mental health and partner involvement into ANC services to develop a holistic approach to maternal healthcare.
